# Global Research Trends and the Association Between Feed Efficiency and Enteric Methane Emissions in Ruminants: A Bibliometric and Thematic Review

**DOI:** 10.3390/ani16182957

**Published:** 2026-09-20

**Authors:** Eduarda Teles Botelho, Karoline Ferreira dos Santos, Lucas Lino Barroso, Maria Júlia Pereira Cordeiro, Leandro Torres de Sousa, Bruna Aparecida Fernandes Guimarães, Hugo Farias Souza, Amanda Rodrigues de Sousa, Giovanni Bárbara Nunes, Lucas Ferreira Gonçalves, Janaina Gomes Araújo Santos, Josiel Ferreira

**Affiliations:** 1 Instituto Federal de Educação, Ciência e Tecnologia Goiano (IF Goiano), Campus Campos Belos, Campos Belos 73840-000, GO, Brazil; eduarda.teles@estudante.ifgoiano.edu.br (E.T.B.); karoline.ferreira@estudante.ifgoiano.edu.br (K.F.d.S.); lucas.lino1@estudante.ifgoiano.edu.br (L.L.B.); cordeiromariajulia90@gmail.com (M.J.P.C.); leandro.torres1@estudante.ifgoiano.edu.br (L.T.d.S.); bruna.guimaraes@estudante.ifgoiano.edu.br (B.A.F.G.); hugo.souza@estudante.ifgoiano.edu.br (H.F.S.); aamandardss@gmail.com (A.R.d.S.); giovanni.nunes@ifgoiano.edu.br (G.B.N.); janaina.santos@ifgoiano.edu.br (J.G.A.S.); 2 Instituto Federal de Educação, Ciência e Tecnologia Goiano (IF Goiano), Campus Rio Verde, Rio Verde 75901-970, GO, Brazil; ferreiralucas1205@gmail.com

**Keywords:** residual feed intake, methane mitigation, rumen microbiome, nutrient utilization, genomic selection, precision phenotyping, sustainability

## Abstract

Enteric methane emissions and feed efficiency are increasingly being investigated as complementary traits for improving the environmental sustainability and productivity of ruminant systems. This study conducted a bibliometric and thematic review of scientific literature published between 2016 and 2025 to characterize research trends and synthesize evidence on the association between feed efficiency and enteric methane emissions in ruminants. Publications retrieved from Scopus and Web of Science were analyzed using Bibliometrix and VOSviewer, including scientific production, collaboration networks, keyword co-occurrence, thematic evolution, and burst analyses. A total of 659 publications from 158 sources were identified, with an annual growth rate of 13.97% and 40.36% international co-authorship. Scientific production increased from 37 publications in 2016 to 120 in 2025. Thematic analyses revealed a transition from the characterization of feed-efficiency and methane traits toward integrated approaches involving methane mitigation, nutrient utilization, rumen microbiology, genomics, and precision phenotyping. Evidence indicates that improved feed efficiency can contribute to lower absolute methane emissions, particularly through reduced feed intake, but does not consistently imply lower methane production relative to feed intake or productive output. Feed efficiency and methane emissions should therefore be considered complementary rather than interchangeable traits when developing sustainable breeding, nutritional, and management strategies.

## Simple Summary

Ruminant livestock provide important foods such as meat and milk but also produce enteric methane, a greenhouse gas generated during feed digestion. Improving feed efficiency—that is, enabling animals to produce the same amount of product while consuming less feed—has been proposed as a strategy to increase productivity while potentially reducing methane emissions. This review examined how scientific research on the relationship between feed efficiency and enteric methane emissions in ruminants developed between 2016 and 2025. A total of 659 publications were analyzed to identify changes in scientific production, collaboration networks, major research topics, and emerging trends, while also examining evidence linking feed efficiency with methane emissions. Research activity increased substantially over the decade and progressively shifted toward integrated approaches involving animal genetics, rumen microorganisms, nutrition, and technologies for measuring animal performance and methane emissions. More feed-efficient animals may produce less methane, particularly because they often consume less feed; however, greater feed efficiency does not always mean lower methane production relative to feed intake or productive output. Therefore, feed efficiency and methane emissions should be considered together when developing strategies for more productive and environmentally sustainable ruminant production systems.

## 1. Introduction

Ruminant production plays an important role in global food security by providing high-quality animal products and contributing to livelihoods and nutritional security worldwide [1,2]. However, improving the sustainability of these systems requires increasing animal productivity and resource-use efficiency while simultaneously reducing their environmental impacts. Among these challenges, enteric methane (CH_4_) emissions and inefficient feed utilization are particularly relevant because they connect animal nutrition and biological efficiency with the environmental footprint of ruminant production systems. Consequently, improving feed efficiency while mitigating enteric methane emissions has become an important strategy for enhancing the productive and environmental sustainability of ruminant production systems [1,2].

Among the greenhouse gases associated with livestock production, methane (CH_4_) from enteric fermentation in ruminants is of particular concern because of its high global warming potential and its status as one of the major sources of emissions from the agricultural sector [3,4,5]. Methane production is an inherent by-product of ruminal fermentation, resulting from the activity of methanogenic microorganisms that utilize hydrogen generated during feed degradation [6,7]. Although this process is essential for maintaining rumen homeostasis, CH_4_ emissions represent a loss of dietary energy that could otherwise be utilized by the animal and contribute substantially to the environmental footprint of meat and milk production [8]. Consequently, developing strategies to mitigate enteric methane emissions without compromising animal productivity has become a major priority in efforts to achieve sustainable livestock intensification.

In this context, feed efficiency has been widely recognized as one of the most important traits for improving the sustainability of livestock production systems [9]. More efficient animals require less feed to achieve the same level of production, thereby reducing feeding costs, improving farm profitability, and potentially decreasing the demand for natural resources and the environmental footprint of animal production [4]. Over the past decades, several feed efficiency indicators have been developed and incorporated into research and genetic improvement programs, including feed conversion ratio (FCR), residual feed intake (RFI), residual gain (RG), and residual intake and gain (RIG) [10,11,12,13]. These metrics have substantially improved the understanding of the biological mechanisms underlying nutrient utilization, energy partitioning, and animal performance in ruminants.

Several studies have investigated the hypothesis that animals with superior feed efficiency also exhibit lower enteric methane emissions [14,15,16,17,18,19]. However, the available evidence remains inconsistent [20], and its interpretation depends on the methane phenotype considered. Absolute methane production, generally expressed as g CH_4_/day, represents the total daily methane output of an animal, whereas methane yield expresses CH_4_ emissions relative to feed intake, and methane intensity expresses emissions relative to productive output. These indicators are therefore biologically and environmentally distinct and should not be interpreted interchangeably. While some studies have reported lower absolute methane production or methane intensity in more feed-efficient animals [15,18,19], others have found weak or no significant associations between feed efficiency and methane-related traits [16,18,21]. A recent systematic review and meta-analysis including 27 studies demonstrated that cattle with low residual feed intake consistently emitted less methane on an absolute basis (g/day); however, no clear differences were observed for several other methane-related variables because of substantial heterogeneity among studies [20]. This variability is likely explained by differences in breed, diet composition, production system, methane measurement methodology, animal category, and the feed efficiency indicator used, highlighting the complexity of the physiological, metabolic, and ruminal microbial mechanisms linking feed efficiency to enteric methanogenesis [11,16,18,20].

In parallel with the increasing number of experimental studies, scientific publications addressing feed efficiency and enteric methane emissions have expanded considerably over the last decade. Previous evidence syntheses have addressed important but distinct components of this research field. A recent bibliometric review characterized scientific trends and advances in residual feed intake and feed efficiency in ruminants [9], whereas a systematic review and meta-analysis specifically evaluated the relationship between residual feed intake and methane-related traits in cattle [20]. However, these approaches differ from the scope of the present review: the former focused broadly on feed efficiency without specifically mapping its intersection with enteric methane emissions, whereas the latter quantitatively synthesized the RFI–methane relationship in cattle without examining the broader bibliometric structure and thematic evolution of this research field. Therefore, an integrated assessment of how research specifically at the interface between feed efficiency and enteric methane emissions has evolved across ruminant species remains warranted.

Accordingly, the present study aimed to characterize the scientific development and thematic evolution of research on the relationship between feed efficiency and enteric methane emissions in ruminants from 2016 to 2025. Specifically, we evaluated publication trends, scientific collaboration networks, and emerging research themes and complemented this bibliometric assessment with a narrative qualitative synthesis of the reported associations between feed efficiency and methane-related traits. This combined approach was intended to identify major patterns, inconsistencies, and knowledge gaps in the literature while maintaining a clear distinction between bibliometric findings and narrative biological interpretation.

## 2. Materials and Methods

### 2.1. Literature Search and Data Collection

**Figure 1:**
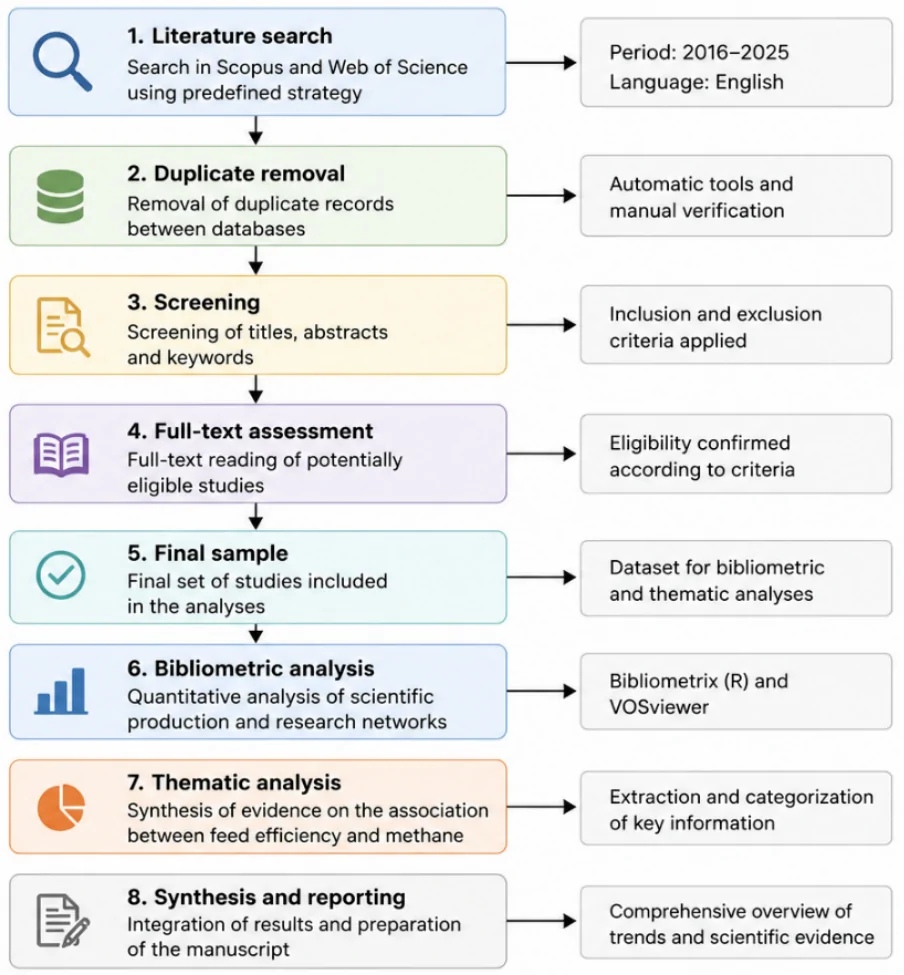
Methodological workflow adopted for the bibliometric and thematic review.

The overall methodological workflow adopted in this study is summarized in Figure 1. The bibliographic search was conducted in the Scopus and Web of Science Core Collection databases, which are recognized as the leading international sources of indexed scientific literature in Animal Science, Agricultural Sciences, and Environmental Sciences.

The search strategy was developed using combinations of descriptors related to feed efficiency, enteric methane emissions, and ruminant species, combined with the Boolean operators AND and OR. The bibliographic searches were conducted on 28 July 2026. The search terms were organized into three complementary conceptual domains: feed-efficiency traits and indicators, methane emissions and methanogenesis, and ruminant species. Alternative terms within each domain were combined using the Boolean operator OR, whereas the three domains were combined using the Boolean operator AND. This structure was designed to retrieve publications addressing the intersection between feed efficiency and enteric methane emissions specifically in ruminants while limiting records focused on these topics independently or on non-ruminant species. Searches were performed in the Title, Abstract, and Keywords fields (TITLE-ABS-KEY) in Scopus and adapted to the Topic (TS) field in the Web of Science Core Collection. The complete search strategy adopted for Scopus is presented in Table 1, while the equivalent syntax was adapted for the Web of Science database.

The search included publications published between 2016 and 2025, without geographical restrictions. Because the bibliographic search was conducted on 28 July 2026, the entire 2025 publication year was covered. Records retrieved from both databases were subsequently checked for duplicates, which were removed before the bibliometric analyses to avoid double counting. The bibliometric dataset included the document types retrieved from Scopus and Web of Science that met the thematic scope of the search, including research articles, reviews, proceedings papers, meeting abstracts, editorial material, corrections, and records classified under combined document types. Records were restricted to publications written in English and published between 2016 and 2025. Duplicate records and publications outside the thematic scope of feed efficiency and enteric methane emissions in ruminants were excluded.

All eligible document types were retained for characterization of the bibliometric landscape. However, the narrative qualitative synthesis of biological associations was restricted to publications providing sufficient primary or synthesized evidence to evaluate the relationship between feed efficiency and methane-related traits.

Following database retrieval and duplicate removal, the remaining records were independently screened by two researchers based on titles, abstracts, and keywords. Records considered potentially eligible were subsequently assessed through full-text reading by the same two researchers. Disagreements regarding study eligibility were resolved through discussion and consensus between the reviewers. At the full-text stage, studies were excluded when they did not simultaneously address feed efficiency and enteric methane emissions in ruminants, did not provide sufficient information to evaluate the relationship between these traits, or fell outside the predefined temporal, language, or thematic scope of the review. The study selection process followed the recommendations of the Preferred Reporting Items for Systematic Reviews and Meta-Analyses (PRISMA) guidelines.

### 2.2. Bibliometric and Thematic Analyses

The bibliographic records retrieved from Scopus and Web of Science were exported in BibTeX and CSV formats for subsequent bibliometric analyses.

Bibliometric analyses were performed using the Bibliometrix package (Biblioshiny interface) [22] in R [23] and VOSviewer software (version 1.6.18) [24]. The following indicators were evaluated: annual scientific production, leading journals, authors, institutions and countries, citation performance, co-authorship networks, keyword co-occurrence, co-citation analysis, bibliographic coupling, and thematic evolution of the research field.

A complementary thematic analysis was conducted to synthesize the scientific evidence regarding the relationship between feed efficiency and enteric methane emissions in ruminants. For each eligible study, information was extracted regarding the ruminant species, production system, feed efficiency indicator (e.g., residual feed intake, feed conversion ratio, residual gain, residual intake and gain, and feed saved), methane measurement method, and the main findings concerning the association between feed efficiency and methane emissions.

Additionally, burst detection analyses of author keywords and co-cited authors were performed in the R environment. Bibliographic metadata were processed using the Bibliometrix package [22], considering the DE field for author keywords and the CR and CR_AU fields to identify the first authors of cited references. Data were cleaned, standardized, and organized using the dplyr, tidyr, and stringr packages [25,26], and synonyms, abbreviations, spelling variants, and inconsistencies in author names and keywords were harmonized prior to analysis.

Annual occurrence series were generated for the period from 2016 to 2025, considering a maximum of one occurrence per document per year. Burst periods were identified using a two-state binomial implementation inspired by Kleinberg’s burst detection algorithm [27]. The ten author keywords with the highest burst strength were selected considering a minimum total frequency of four occurrences, whereas the fifteen co-cited authors with the highest burst strength were selected considering a minimum frequency of eight citing documents. Visualizations were produced using the ggplot2 package [25], and the numerical outputs together with the analysis parameters were exported to Microsoft Excel spreadsheets using the openxlsx package [26].

The extracted information was organized into thematic categories to identify research trends, areas of consensus, conflicting findings, and knowledge gaps reported over the last decade. The thematic analysis was conducted through a narrative qualitative synthesis of the selected studies. The articles were grouped according to predefined descriptive categories based on the information extracted from each study, including feed efficiency indicators, methane measurement methods, ruminant species, production systems, and the reported relationship between feed efficiency and methane emissions. This component was intended to provide a structured narrative interpretation of the retrieved literature rather than a formal systematic evidence synthesis.

## 3. Global Research Trends on Feed Efficiency and Enteric Methane Emissions

### 3.1. Annual Scientific Production

A total of 659 publications were retrieved between 2016 and 2025, demonstrating a marked expansion in scientific research addressing the relationship between feed efficiency and enteric methane emissions in ruminants (Table 2). The literature showed an average annual growth rate of 13.97%, with an average of 26.24 citations per document, indicating both the rapid expansion and the scientific relevance of this research field. Overall, the dataset comprised publications from 158 scientific sources, predominantly original research articles, which accounted for approximately 81% of all documents, followed by review papers.

**Figure 2:**
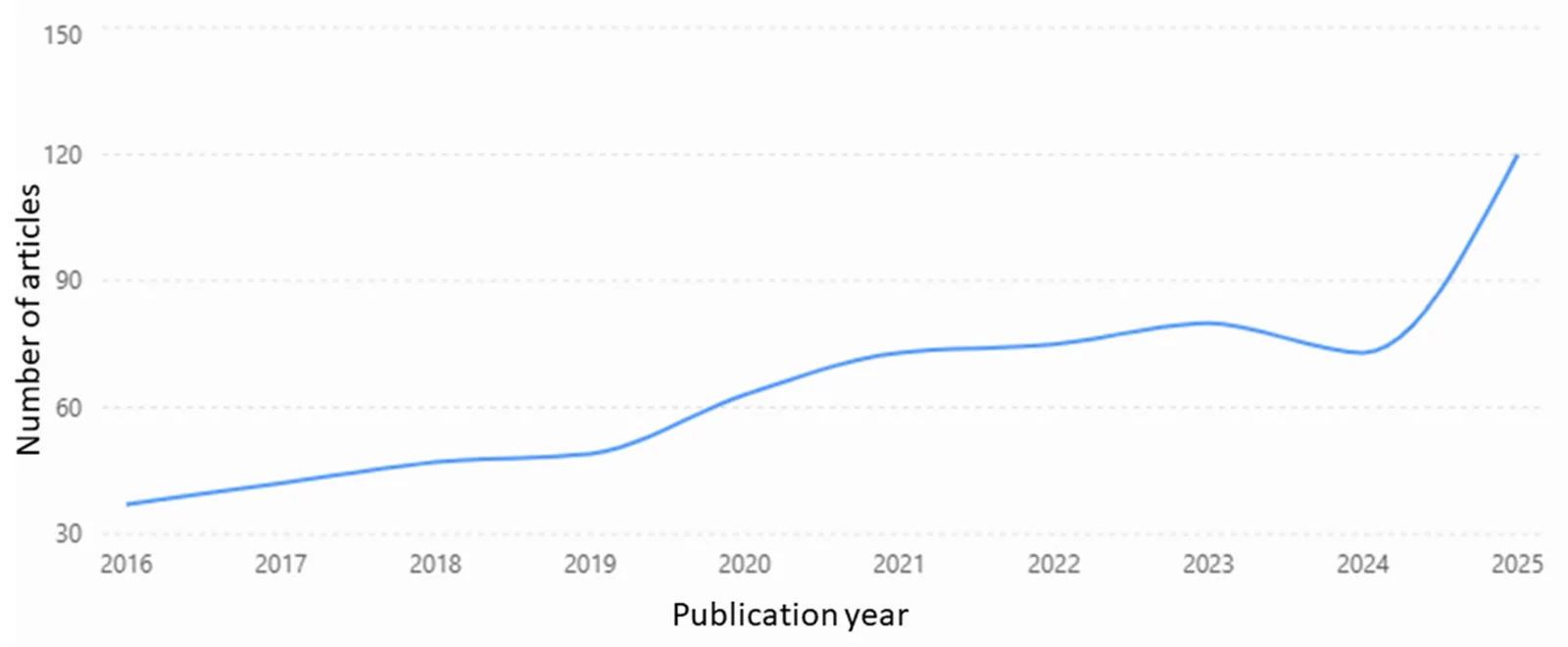
Annual scientific production on feed efficiency and enteric methane emissions in ruminants (2016–2025).

Annual publication output showed a consistent upward trend throughout the study period (Figure 2). The number of publications increased from 37 in 2016 to 120 in 2025, representing more than a threefold increase over the decade. Although publication output remained relatively stable between 2021 and 2024, ranging from 73 to 80 articles per year, a pronounced increase was observed in 2025, suggesting renewed momentum in research activity. This pattern reflects the growing international interest in improving feed efficiency while simultaneously mitigating enteric methane emissions, driven by increasing environmental concerns, advances in precision livestock technologies, genomic selection programs, and the global demand for more sustainable livestock production systems.

The continuous growth observed over the last decade also indicates that feed efficiency and methane mitigation have evolved from relatively independent research topics into increasingly integrated areas of investigation. This convergence is consistent with the recognition that improving biological efficiency represents one of the most promising strategies for simultaneously enhancing animal productivity and reducing the environmental footprint of ruminant production systems. The recent acceleration in publication output further suggests that this field will likely remain an important research priority in the coming years.

### 3.2. General Characteristics of the Retrieved Literature

The final dataset comprised 659 publications indexed in 158 scientific sources between 2016 and 2025 (Table 2). The retrieved literature showed an average annual growth rate of 13.97%, with a mean document age of 4.58 years and an average of 26.24 citations per document, reflecting the rapid expansion and citation impact of research on feed efficiency and enteric methane emissions in ruminants. A total of 1406 author keywords and 1419 Keywords Plus were identified, indicating a broad diversity of research topics and terminology within the field.

Scientific production was dominated by original research articles (536 documents), whereas review papers accounted for 89 publications, demonstrating that experimental research remains the primary driver of knowledge generation in this area. Collaboration among researchers was substantial, with an average of 6.67 co-authors per publication and 40.36% of the studies involving international collaboration. In contrast, only 11 publications were produced by a single author, highlighting the multidisciplinary and collaborative nature of research on feed efficiency and enteric methane emissions.

### 3.3. Most Productive Authors, Institutions, Countries and Journals

Scientific production on feed efficiency and enteric methane emissions in ruminants is concentrated among a relatively small group of highly productive researchers, reflecting the existence of well-established international research networks. Among the most prolific authors, L. Guan ranked first with 21 publications, followed by F. Miglior (15 publications), J. Pryce (14), S. Waters (13), and J. Lassen, P. Løvendahl, X. Zhang, and R. Roehe, each contributing 12 publications (Table 3). Other authors with substantial scientific output included C. Richardson and D. Kenny (11 publications each), P. Huhtanen, Y. Wang, and P. Amer (10 publications each), highlighting the relatively concentrated distribution of scientific production within the field.

When fractionalized authorship was considered, which accounts for the relative contribution of each author to multi-authored publications, L. Guan remained the leading contributor (5.30), followed by C. Richardson (2.60), P. Huhtanen (2.57), P. Løvendahl (2.25), and J. Lassen (2.23). Although several authors published a large number of papers, the lower fractionalized values observed for most researchers indicate that advances in this field have largely resulted from collaborative, multi-institutional research efforts rather than individual research groups.

The predominance of collaborative authorship reflects the multidisciplinary nature of research on feed efficiency and enteric methane emissions, which integrates expertise in animal breeding, nutrition, rumen microbiology, genetics, precision livestock farming, and environmental sciences. Such collaboration has been fundamental for advancing knowledge on the biological mechanisms underlying feed efficiency and methane production, as well as for developing strategies aimed at improving livestock productivity while reducing greenhouse gas emissions.

### 3.4. Collaboration Networks

**Figure 3:**
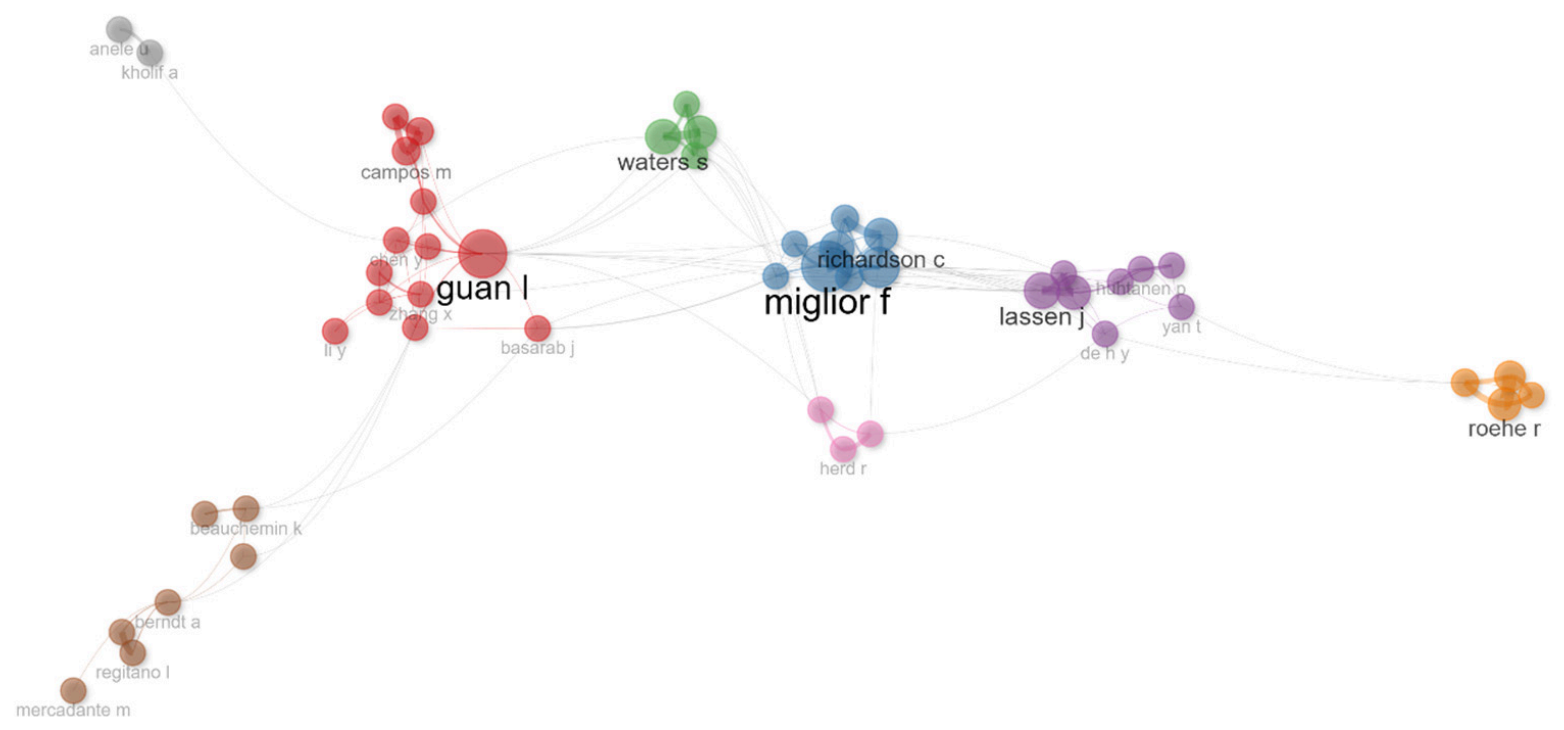
Co-authorship network of authors publishing on feed efficiency and enteric methane emissions in ruminants.

The co-authorship network revealed a highly collaborative research landscape characterized by several interconnected groups of authors working on feed efficiency and enteric methane emissions in ruminants (Figure 3). Rather than being concentrated around a single research group, the scientific output was distributed among multiple collaborative clusters, indicating the existence of complementary research teams with expertise in animal breeding, nutrition, rumen microbiology, genetics, methane measurement technologies, and precision livestock farming. The extensive connectivity among authors suggests that scientific advances in this field have largely resulted from long-term collaborations and multidisciplinary approaches rather than isolated research efforts.

**Figure 4:**
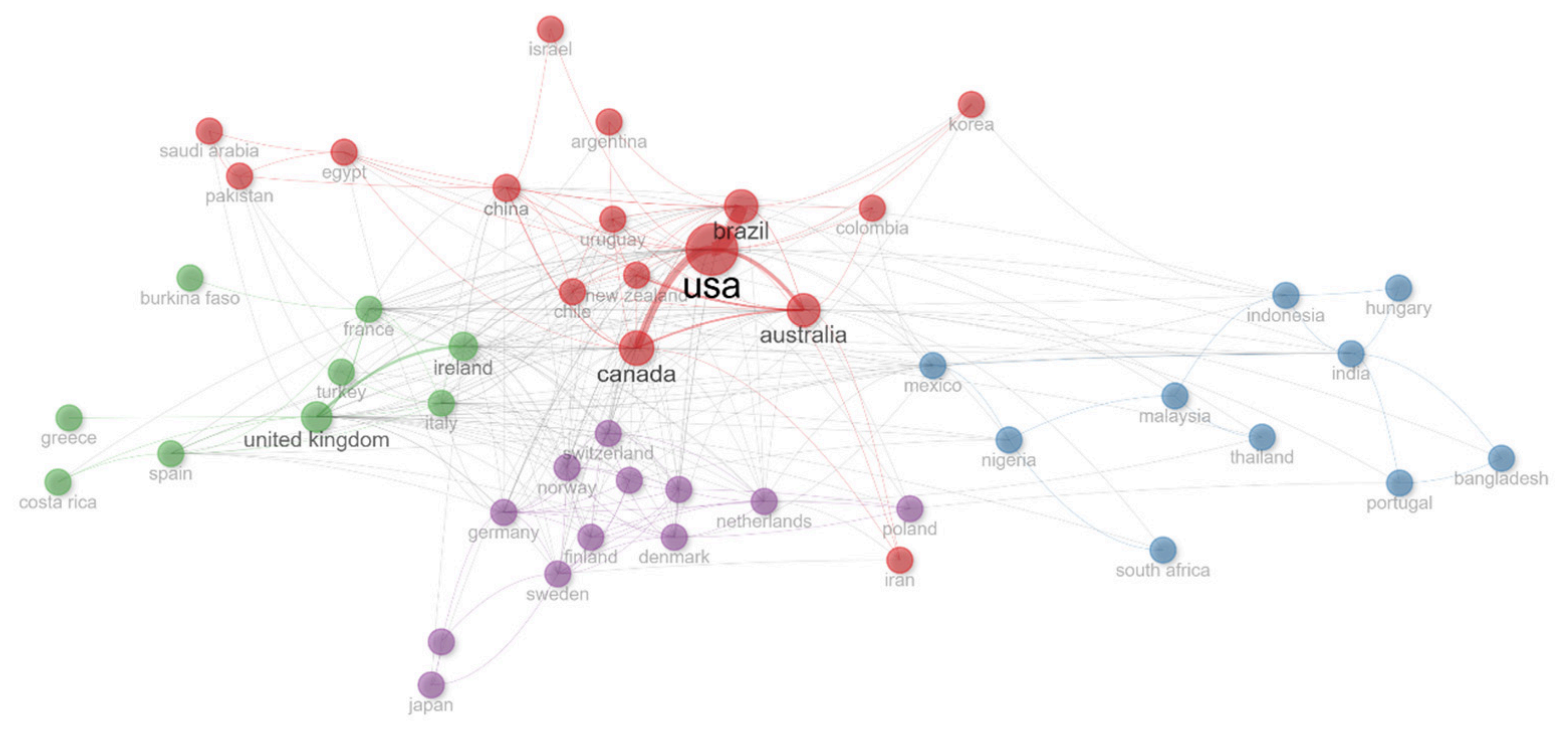
International collaboration network among countries contributing to research on feed efficiency and enteric methane emissions in ruminants.

The international collaboration network further demonstrated that research on feed efficiency and enteric methane emissions has developed through strong partnerships among countries with established livestock industries (Figure 4). These collaborative relationships facilitate the sharing of experimental infrastructure, methane phenotyping platforms, genomic resources, and expertise in animal breeding and nutrition, thereby accelerating scientific progress and improving the robustness and comparability of research findings. The substantial proportion of internationally co-authored publications observed in the dataset (40.36%; Table 2) reinforces the increasingly global nature of research in this field.

The collaborative structure identified in both author and country networks highlights the increasing integration of expertise across disciplines and geographic regions. Such international cooperation has become particularly important for addressing the biological and environmental complexity associated with feed efficiency and enteric methane emissions, enabling the development of standardized methodologies, large-scale datasets, and coordinated research initiatives aimed at improving the sustainability of ruminant production systems.

### 3.5. Research Themes and Keyword Analysis

**Figure 5:**
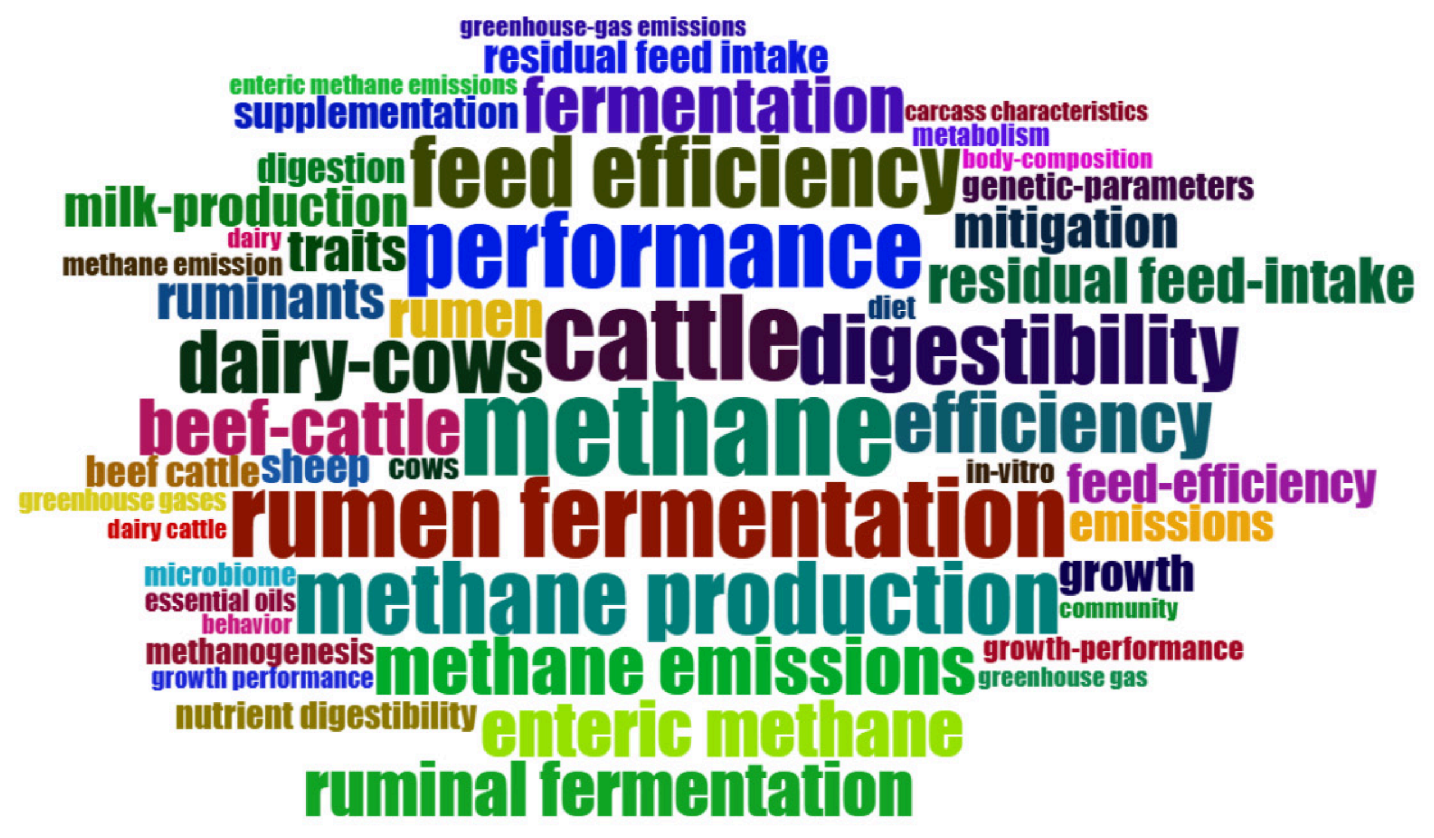
Word cloud of author keywords on feed efficiency and enteric methane emissions in ruminants (2016–2025).

The analysis of author keywords revealed the main scientific themes that have shaped research on feed efficiency and enteric methane emissions in ruminants over the last decade. The word cloud (Figure 5) illustrates the predominance of terms directly related to methane emissions, feed efficiency, residual feed intake, cattle, rumen microbiology, and greenhouse gas mitigation, highlighting the close integration between animal productivity and environmental sustainability within this research field. The high frequency of these keywords demonstrates that improving feed utilization while reducing methane emissions has become one of the principal objectives of contemporary ruminant research.

**Figure 6:**
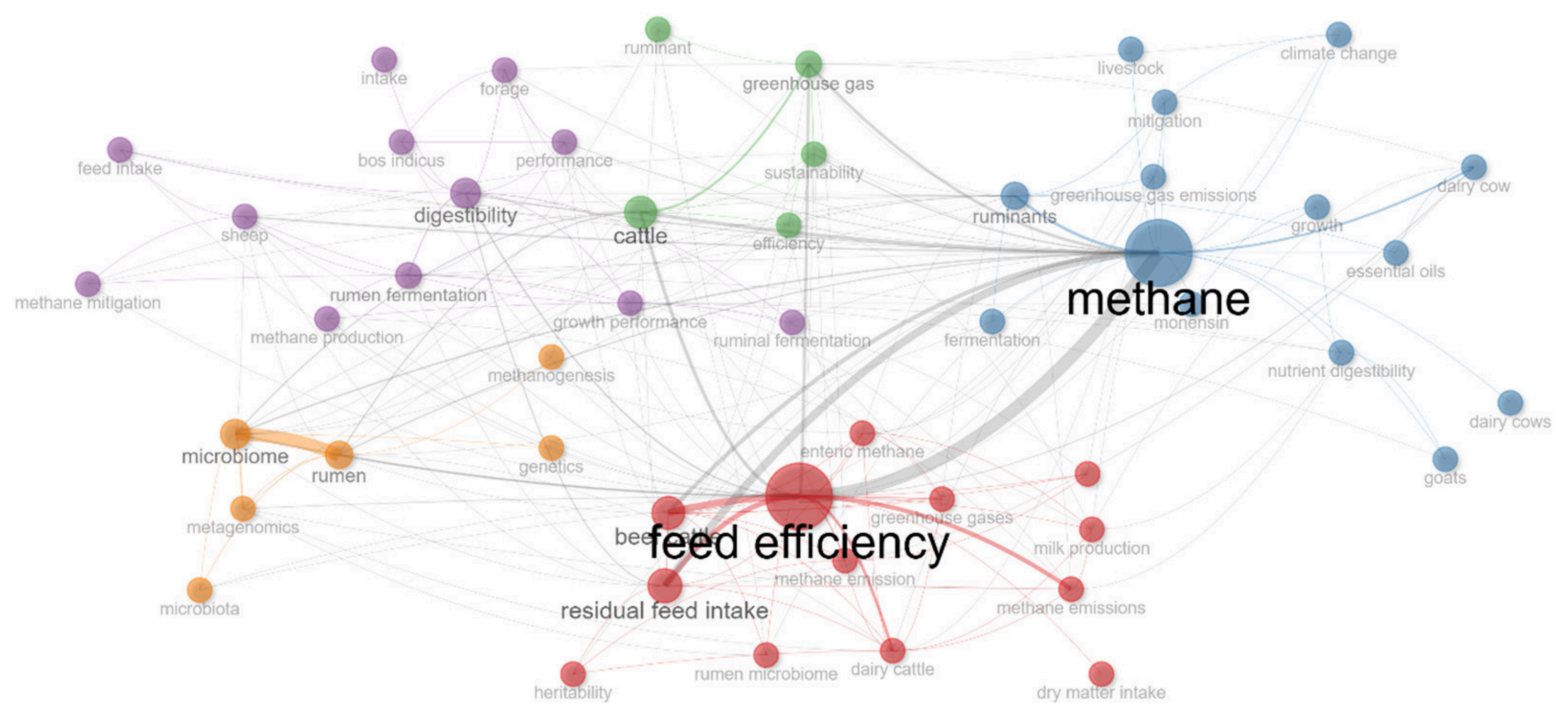
Keyword co-occurrence network in studies on feed efficiency and enteric methane emissions in ruminants.

The keyword co-occurrence network (Figure 6) further demonstrated that the literature is organized around several interconnected research themes rather than isolated topics. Feed efficiency indicators, particularly residual feed intake, were strongly associated with methane emissions, rumen fermentation, animal performance, microbial ecology, and genetic improvement, indicating that these areas have increasingly converged into multidisciplinary research programs. The extensive interconnections among keywords also suggest that nutritional, genetic, physiological, and microbiological approaches are being integrated to better understand the biological mechanisms linking feed efficiency and enteric methane production.

**Figure 7:**
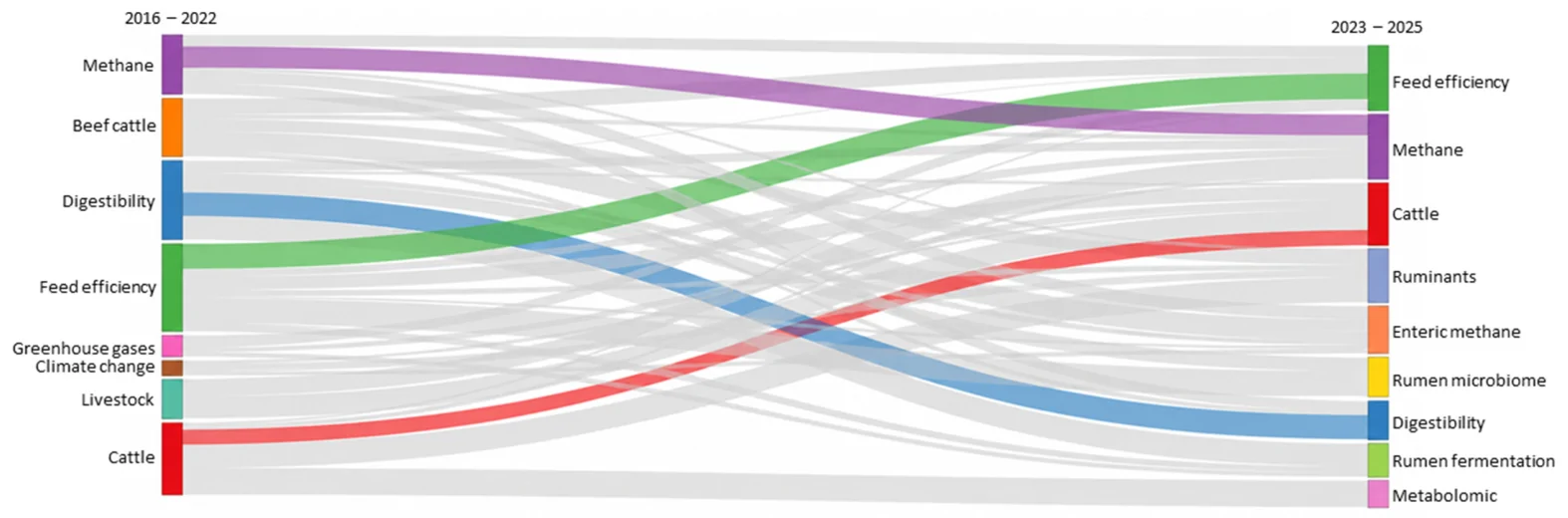
Thematic evolution of research on feed efficiency and enteric methane emissions in ruminants.

The thematic evolution analysis (Figure 7) revealed a progressive shift in research priorities throughout the study period. Earlier studies were primarily focused on describing feed efficiency traits and methane emissions independently, whereas more recent investigations have increasingly incorporated genomic selection, rumen microbiome characterization, precision livestock farming, and methane mitigation strategies. This evolution reflects the maturation of the research field, moving from descriptive investigations toward more integrated approaches that combine animal breeding, nutrition, microbiology, and environmental sustainability to develop more efficient and climate-resilient ruminant production systems.

### 3.6. Emerging Research Trends: Burst Analyses of Author Keywords and Co-Cited Authors

Burst detection analysis revealed important temporal changes in research priorities over the last decade (Table 4). Early research activity was characterized by strong citation bursts for broad descriptors such as methane, reflecting the initial emphasis on quantifying enteric methane emissions and understanding their relationship with feed efficiency. As the field matured, keywords associated with animal performance and biological efficiency, including residual feed intake, average daily gain, and efficiency, gained prominence, indicating a growing interest in integrating productivity traits with environmental performance. More recently, emerging bursts for terms such as methane mitigation, nutrient utilization, and beef cattle suggest a shift toward developing practical mitigation strategies, improving nutrient-use efficiency, and expanding research under commercial production conditions. The appearance of metagenomics among the strongest burst keywords further highlights the increasing application of high-throughput molecular approaches to investigate the role of the rumen microbiome in feed efficiency and methane production.

The analysis of co-cited author bursts demonstrated that the evolution of this research field has been strongly influenced by a relatively small group of highly cited researchers (Table 5). Early citation bursts were dominated by authors such as Pinares-Patiño, Herd, Hegarty, and Nkrumah, whose studies established the conceptual foundations relating feed efficiency, residual feed intake, and enteric methane emissions. During subsequent years, authors including Flay, Kelly, McDonnell, and Knapp emerged as influential contributors, reflecting advances in methane phenotyping, genetic evaluation, and nutritional strategies. More recently, the burst associated with Wang and the renewed prominence of Kholif indicate growing scientific interest in novel nutritional interventions, precision livestock technologies, and integrated approaches for mitigating methane emissions while maintaining or improving feed efficiency.

Together, the keyword and co-cited author burst analyses demonstrate that research has evolved from predominantly descriptive studies toward increasingly multidisciplinary investigations integrating genetics, nutrition, rumen microbiology, precision livestock farming, and environmental sustainability. These temporal patterns indicate that future advances will likely depend on combining genomic, microbial, nutritional, and precision phenotyping approaches to develop more efficient and climate-smart ruminant production systems.

## 4. Discussion

The findings of this review are interpreted at three complementary levels. First, bibliometric indicators describe the structure and evolution of the retrieved literature, including publication trends, collaboration networks, and thematic patterns. Second, the narrative qualitative synthesis summarizes the biological associations between feed efficiency and methane-related traits reported in the selected studies. Third, these findings are discussed in the context of the broader literature to explore their biological and practical implications. These levels of interpretation are distinguished throughout the Discussion to avoid attributing biological conclusions directly to bibliometric patterns.

### 4.1. Expansion and Consolidation of Research on Feed Efficiency and Enteric Methane Emissions

The marked increase in scientific production observed between 2016 and 2025 demonstrates that the relationship between feed efficiency and enteric methane emissions has become an increasingly relevant research topic in ruminant production. The more than threefold increase in annual publications, together with the annual growth rate of 13.97%, indicates not only an expansion in the volume of research but also the progressive consolidation of a scientific field in which productive efficiency and environmental sustainability are increasingly investigated as interconnected objectives. This pattern is consistent with recent bibliometric evidence showing substantial growth in research on residual feed intake and feed efficiency in ruminants and an increasing integration of these traits with methane emissions, sustainability, genetics, nutrition, and rumen biology [9].

This expansion likely reflects a broader change in the objectives of ruminant production research. Feed efficiency was traditionally investigated primarily because of its direct relationship with feed costs and animal performance. However, the increasing importance of greenhouse gas mitigation has expanded this perspective, as improving the efficiency with which animals convert feed resources into animal products may simultaneously contribute to economic and environmental sustainability [4,9]. Enteric methane represents both an important source of greenhouse gas emissions from ruminant systems and a loss of dietary energy during ruminal fermentation. Consequently, the simultaneous evaluation of feed utilization and methane production provides a biologically and environmentally relevant framework for identifying animals and production systems capable of producing more animal products with lower resource use and potentially lower environmental impact [5,7,8].

The acceleration of scientific production should also be interpreted in the context of recent methodological and technological developments. Individual measurements of feed intake and methane emissions are technically demanding and costly, particularly when large populations are required for genetic evaluation. The increasing availability of automated feeding systems, GreenFeed systems, respiration chambers, portable accumulation chambers, genomic information, and large multicountry datasets has progressively increased the capacity to phenotype animals for both feed efficiency and methane-related traits [14]. These developments have facilitated the incorporation of such phenotypes into nutritional, physiological, microbial, and genetic studies and have contributed to moving the field from relatively isolated evaluations of feed efficiency or methane emissions toward integrated assessments of animal efficiency and environmental performance.

Nevertheless, the increase in publication output should not be interpreted as evidence that the biological relationship between feed efficiency and methane emissions has been fully resolved. Rather, the rapid expansion of the field has revealed considerable complexity in this association. Differences among feed efficiency indicators, methane metrics, diets, breeds, production stages, and measurement methodologies can substantially affect the magnitude and even the direction of the relationships reported among studies. Thus, the consolidation of this research field has been accompanied by a transition from the relatively simple hypothesis that more feed-efficient animals necessarily emit less methane toward a more comprehensive investigation of how feed intake, digestion, rumen fermentation, host genetics, and microbial ecology interact to determine both productive efficiency and methane emissions [16,20,21]. This transition provides an important basis for interpreting the thematic evolution and emerging research priorities identified in the present bibliometric analysis.

### 4.2. Scientific Collaboration and International Research Networks

The high level of scientific collaboration identified in this review highlights the multidisciplinary and international nature of research addressing feed efficiency and enteric methane emissions. More than 40% of the publications involved international co-authorship, while the low number of single-authored documents and the extensive connectivity observed in the co-authorship network indicate that knowledge generation in this field is largely supported by collaborative research structures. Rather than being dominated by a single research group, the network comprised several interconnected clusters, suggesting that advances have emerged from complementary groups specializing in animal nutrition, genetics and breeding, rumen microbiology, methane phenotyping, and precision livestock technologies.

This collaborative structure is particularly relevant because simultaneous phenotyping of feed efficiency and enteric methane emissions remains technically demanding and resource-intensive. Accurate assessment of feed efficiency requires reliable individual feed intake and performance records, whereas methane phenotyping depends on specialized measurement approaches, including respiration chambers, GreenFeed systems, portable accumulation chambers, and other indirect or direct measurement techniques. Although respiration chambers provide highly controlled measurements, their cost, labor requirements, and relatively low animal throughput limit their application to large populations. Alternative systems have increased phenotyping capacity, but differences among measurement protocols, environments, diets, and methane traits can affect the comparability of observations. Consequently, collaborative initiatives that combine experimental infrastructure and datasets across research centers are particularly valuable for increasing sample sizes and improving the robustness of genetic, nutritional, and biological evaluations.

International cooperation is also important for expanding the environmental and genetic diversity represented in methane and feed-efficiency studies. Relationships among feed intake, methane production, animal performance, and feed efficiency may depend on breed, diet, production system, physiological stage, and environmental conditions [14,15,16,17,20]. Therefore, evidence generated within a single population or production environment may not necessarily be transferable to other ruminant systems. The integration of data from different populations and research environments provides opportunities to evaluate the consistency of methane-related phenotypes and their associations with feed efficiency across breeds and management conditions, while also supporting the development of more representative genetic parameters and selection strategies.

Overall, the collaboration networks identified in this review indicate that research on feed efficiency and enteric methane emissions is organized around interconnected international groups with complementary expertise and infrastructure. Such collaboration is particularly relevant for studies requiring large-scale phenotyping and integration of nutritional, genomic, microbial, and environmental information. However, the interpretation and integration of multicenter data depend on adequate harmonization of phenotypes and measurement protocols, particularly because methane production, methane yield, methane intensity, and residual methane represent distinct biological traits.

### 4.3. Evolution of Research Themes: From Phenotypic Efficiency to Integrated Approaches

The thematic patterns identified in this review indicate that research on feed efficiency and enteric methane emissions has undergone a substantial conceptual transition over the last decade. Although feed efficiency and methane remain central components of the scientific network, their strong connections with rumen fermentation, animal performance, microbial ecology, and genetic improvement demonstrate that these traits are increasingly being investigated within a common biological framework. This evolution suggests that the field has progressed beyond the characterization of individual phenotypes toward understanding the mechanisms that jointly determine nutrient utilization, productive performance, and methane production.

Residual feed intake occupies a particularly important position in this transition. Unlike conventional feed conversion measures, RFI evaluates variation in feed intake after accounting for expected requirements for maintenance and production, thereby providing a phenotype that is less directly dependent on production level. Its prominence in the keyword analyses reflects its widespread use in studies attempting to identify biological differences between animals with contrasting efficiencies. However, the relationship between RFI and methane production is not necessarily straightforward. More efficient animals frequently consume less feed for a similar level of production, which can reduce absolute methane output, but this does not necessarily imply lower methane production per unit of feed consumed. This distinction has encouraged a shift from comparisons based solely on daily methane production toward the simultaneous evaluation of methane production, yield, intensity, and residual methane phenotypes.

The increasing prominence of rumen fermentation and microbial ecology further demonstrates the search for biological explanations underlying variation in both traits. Enteric methane production is intrinsically linked to ruminal fermentation because methanogenic archaea utilize reducing equivalents, particularly hydrogen, generated during microbial degradation of feed [6,7]. Differences among animals in feed intake, passage rate, digestibility, fermentation pathways, and microbial community structure may therefore contribute simultaneously to variation in feed efficiency and methane emissions [6,11]. Consequently, the rumen microbiome has increasingly been investigated as a potential intermediate phenotype linking diet, host genetics, nutrient utilization, and methanogenesis. The emergence of metagenomics in the burst analysis reinforces this mechanistic transition by indicating increasing use of high-throughput molecular approaches to characterize microbial communities and their functional potential.

The growing integration of genomic information represents another important stage in the development of the field. Both feed efficiency and methane-related traits exhibit biological variation among animals, creating opportunities for genetic improvement when reliable and sufficiently large phenotypic datasets are available. Advances in genomic selection have made it possible to investigate genetic relationships among feed intake, productive performance, and methane phenotypes and to consider environmental traits within breeding objectives [18,19,21]. However, selection for reduced methane emissions cannot be considered independently of productive performance and feed utilization. Genetic strategies should therefore seek animals that combine favorable efficiency with reduced environmental impact while avoiding undesirable correlated responses in economically important traits.

The temporal patterns detected by the burst analysis also suggest a gradual transition from understanding the problem toward developing applicable solutions. Earlier citation bursts associated with methane and residual feed intake reflect efforts to establish and characterize the relevant phenotypes, whereas the more recent emergence of terms such as methane mitigation, nutrient utilization, and efficiency indicates increasing emphasis on intervention and implementation. The recent prominence of beef cattle is also relevant because it suggests expansion of research toward production contexts in which individual phenotyping can be particularly challenging, especially under grazing and commercial conditions. Thus, methodological advances that permit reliable measurements outside highly controlled experimental environments will be essential for translating biological knowledge into practical mitigation strategies.

Taken together, the thematic evolution observed in this review suggests that feed efficiency and methane mitigation are no longer being treated as isolated objectives. The emerging research framework integrates host genetics, rumen microbiology, nutrition, animal physiology, and precision phenotyping to explain individual variation and identify animals and management strategies capable of combining productivity with lower environmental impact. Importantly, this integration also indicates that no single biological mechanism or mitigation strategy is likely to explain the relationship between feed efficiency and methane across all production systems. Future progress will therefore depend on multidisciplinary approaches capable of distinguishing reductions in methane that result primarily from lower feed intake from those associated with genuine changes in ruminal fermentation, nutrient-use efficiency, or methane production independent of intake.

### 4.4. Biological Relationship Between Feed Efficiency and Enteric Methane Emissions

The increasing integration between feed efficiency and enteric methane observed in the bibliometric and thematic analyses is biologically supported by the fact that both traits are closely associated with feed intake, nutrient digestion, energy metabolism, and rumen fermentation [14,16,18,20]. Nevertheless, the available evidence does not support a simple assumption that more feed-efficient animals invariably have a lower intrinsic capacity for methane production. Instead, the relationship depends strongly on how feed efficiency and methane emissions are expressed, as well as on the biological mechanisms responsible for differences among animals. This distinction is essential when interpreting the potential of feed efficiency as an indirect strategy for reducing the environmental impact of ruminant production.

Among feed-efficiency traits, RFI has received particular attention because it identifies animals that consume more or less feed than expected for their maintenance and production requirements. Low-RFI animals are therefore considered more feed efficient and generally consume less dry matter than high-RFI animals while maintaining comparable levels of production. Because dry matter intake is a major determinant of daily enteric methane production, reduced intake provides a direct mechanism through which more efficient animals may produce less CH_4_ per day [4,14,15,20]. Indeed, the available evidence summarized in the literature indicates that low-RFI cattle can exhibit lower absolute methane emissions [14,15,16,17,18,20,21]. However, when methane production is expressed relative to dry matter intake, the association with feed efficiency becomes considerably less consistent. Thus, part of the apparent environmental advantage associated with low RFI may arise from reduced feed consumption rather than from an intrinsic reduction in methane formation per unit of substrate fermented.

This distinction emphasizes the importance of the methane phenotype used to evaluate environmental efficiency. Methane production expressed as g CH_4_/day quantifies the animal’s absolute contribution to emissions, whereas methane yield, commonly expressed relative to dry matter intake, provides information on methane production per unit of feed consumed. Methane intensity expresses emissions relative to an output such as body weight gain or milk production and therefore incorporates productive performance into the assessment. These phenotypes answer different biological and environmental questions and should not be interpreted interchangeably. An animal may emit less methane per day because it consumes less feed, while presenting similar or even greater methane yield. Conversely, an animal with relatively high daily methane production may have lower methane intensity if greater emissions are accompanied by substantially greater production. Therefore, identifying animals with favorable environmental performance requires simultaneous consideration of absolute emissions, feed intake, and productive output.

The emergence of residual methane traits represents an important development in this context. Conceptually, residual methane phenotypes attempt to describe individual variation in methane production after accounting for major determinants such as feed intake and, depending on the definition adopted, production or body size. Such approaches may help distinguish animals that emit less methane simply because they consume less feed from those that produce less methane than expected for their level of intake and performance. This distinction is particularly relevant for breeding programs because reducing methane through lower feed intake alone may not represent the same biological mechanism or provide the same long-term opportunities as selecting animals with inherently lower methane production while maintaining productivity. The appearance of residual methane among the emerging themes identified in the present review therefore reflects a broader effort to develop phenotypes that better separate environmental efficiency from differences in intake and production.

Beyond feed intake, several mechanisms may contribute to the relationship between feed efficiency and methane production. Variation in digestibility, ruminal passage rate, feeding behavior, maintenance energy requirements, tissue metabolism, and fermentation patterns have been associated with differences in feed efficiency among individual animals. Changes in these processes can influence the amount of fermentable substrate available in the rumen and the production and utilization of hydrogen, thereby affecting methanogenesis [4,7,9,16]. For example, changes in volatile fatty acid profiles that favor propionate formation may provide an alternative hydrogen sink and reduce the availability of reducing equivalents for methane formation [7]. Conversely, greater fiber digestion may increase substrate availability for fermentation and potentially favor acetate production and methanogenesis. Consequently, differences in digestive and fermentation characteristics can contribute to variation in methane emissions independently of, or in interaction with, differences in feed intake.

The rumen microbiome adds another level of complexity to this relationship. Methane formation results primarily from the activity of methanogenic archaea, but their activity depends on metabolic interactions with other members of the rumen microbial community that generate hydrogen and other substrates during fermentation [6,7,11]. Therefore, variation in methane production cannot necessarily be explained solely by differences in methanogen abundance. Differences in microbial community structure, metabolic pathways, substrate availability, and interactions between hydrogen-producing and hydrogen-utilizing microorganisms may all contribute to individual variation in methane phenotypes [6,7,11]. The growing prominence of rumen microbiology and metagenomics identified in the present bibliometric analysis is consistent with the increasing recognition that microbial function, rather than taxonomic composition alone, may be necessary to explain why animals with apparently similar intake and performance can differ in methane emissions and feed efficiency [11,18,19,21].

Host genetics may further contribute to this individual variation by influencing feed intake, digestion, rumen physiology, passage rate, and the ecological conditions under which microbial communities develop. Consequently, feed efficiency, methane production, and rumen microbial characteristics should not necessarily be considered independent biological components. Rather, they represent interacting phenotypes within a complex host–microbiome–diet system [11,18,19,21]. This perspective helps explain the growing convergence of genetics, microbiology, nutrition, and precision phenotyping observed in the thematic analyses and supports the development of integrated approaches in which methane mitigation is evaluated alongside feed efficiency and economically important production traits. For example, recent studies have jointly investigated methane- and feed-efficiency-related phenotypes in association with rumen microbial profiles and host genomic information [28,29], illustrating the potential of integrative approaches to characterize the biological mechanisms underlying variation in these complex traits.

Overall, the literature reviewed here suggests that improved feed efficiency may contribute to reducing the environmental footprint of ruminant production, particularly when improved efficiency is associated with lower feed requirements for a given level of animal output. However, feed efficiency should not be considered a universal proxy for low enteric methane emissions. The magnitude and direction of the relationship depend on the efficiency indicator, methane phenotype, intake level, diet, animal population, and underlying digestive and microbial mechanisms [16,18,20,21]. Future selection and mitigation strategies should therefore prioritize animals that combine favorable feed efficiency with genuinely favorable methane phenotypes rather than assuming that selection for one trait will automatically produce an equivalent response in the other. Simultaneous phenotyping of feed intake, productive performance, and multiple methane metrics will be essential for distinguishing improvements resulting from reduced resource use from those associated with intrinsic reductions in methanogenesis.

### 4.5. Sources of Heterogeneity in the Relationship Between Feed Efficiency and Methane Emissions

The variability in the relationships reported between feed efficiency and enteric methane emissions is likely attributable to the interaction of methodological, nutritional, genetic, and physiological factors. Differences among studies should therefore not necessarily be interpreted as contradictory evidence, but rather as an indication that the association between these traits is context dependent. In particular, the feed efficiency indicator and methane phenotype adopted, diet composition, animal genotype and physiological stage, production system, and methane measurement methodology can substantially influence the magnitude and direction of the observed associations [16,17,20,21].

An important source of heterogeneity is the definition of feed efficiency itself. Residual feed intake, feed conversion ratio, residual gain, residual intake and gain, and related indicators quantify different aspects of biological efficiency and differ in their mathematical relationships with feed intake and production. Residual feed intake, for example, is designed to be phenotypically independent of the production traits included in its prediction model, whereas feed conversion ratio is directly influenced by both feed intake and productive output [4,9,10]. Consequently, animals classified as efficient according to one indicator may not necessarily occupy the same ranking when another metric is applied. Comparisons among studies using different definitions of efficiency should therefore be made cautiously, particularly when attempting to infer their relationships with methane emissions.

A similar issue applies to the definition of the methane phenotype. As discussed above, methane production, methane yield, methane intensity, and residual methane represent distinct dimensions of environmental performance. Because feed intake strongly influences daily methane production, studies comparing absolute CH_4_ emissions may detect differences between feed-efficiency groups that become weaker or disappear when emissions are adjusted for dry matter intake. Likewise, expressing methane relative to animal products can lead to different conclusions depending on differences in growth rate or milk production [14,18,20,21]. Therefore, apparently inconsistent findings may partly reflect differences in the denominator or statistical adjustment used to express methane emissions rather than genuine biological disagreement among studies [20].

Diet composition represents another major source of variation. The amount and type of carbohydrate fermented in the rumen influence fermentation pathways, hydrogen production, volatile fatty acid profiles, digestibility, passage rate, and ultimately methane formation [7,16]. Diets differing in forage-to-concentrate ratio, fiber concentration, starch availability, forage quality, lipid content, or feed additives can therefore modify both feed efficiency and methane production [8,16]. Importantly, the biological differences between efficient and inefficient animals may also depend on the nutritional environment in which they are evaluated. Relationships observed under high-concentrate finishing diets, for example, may not be directly transferable to forage-based or grazing systems [15,16,17]. This diet-by-animal interaction limits the generalization of results across experimental conditions and reinforces the need to evaluate efficiency and methane phenotypes under production environments representative of their intended application.

Animal-related factors further contribute to heterogeneity. Species, breed, age, sex, physiological stage, mature body size, and production level influence nutrient requirements, voluntary feed intake, digestive kinetics, and rumen fermentation [4,9,20]. Genetic differences among populations may also affect the magnitude of variation available for feed efficiency and methane-related traits [18,19,21]. As a consequence, associations detected in growing beef cattle cannot automatically be extrapolated to lactating dairy cows, sheep, goats, or animals maintained under different environmental and nutritional conditions. The predominance of cattle-related terms identified in the present thematic analyses also indicates that the current evidence base may be more representative of bovine production systems than of other ruminant species.

Methane measurement methodology is an additional source of variation among studies. Respiration chambers, GreenFeed systems, portable accumulation chambers, tracer techniques, and other approaches differ in measurement duration, frequency, animal behavior during measurement, environmental control, and ability to capture diurnal variation in methane production. Measurements obtained over short periods or from relatively few visits may be particularly sensitive to variation in feeding behavior and temporal patterns of ruminal fermentation [30]. Conversely, highly controlled methods can provide precise measurements but may alter normal feeding behavior or differ from commercial production conditions. Methodological differences can therefore contribute to variation in methane estimates and reduce direct comparability among studies, particularly when different techniques are used to derive phenotypes intended for genetic or biological evaluation.

Production environment also deserves consideration. Many detailed studies of feed efficiency and methane production require individual feed-intake measurements and specialized methane-monitoring equipment and are therefore conducted under controlled experimental conditions [14,16]. Although these designs provide high-quality phenotypes, they may not fully represent the variability encountered under grazing or commercial production systems [15,17]. Under field conditions, differences in forage availability and quality, climate, supplementation, competition, activity, and grazing behavior may modify both energy expenditure and nutrient intake. Expanding phenotyping under commercial and pasture-based conditions is therefore important for determining whether relationships identified in controlled environments remain consistent at the production system level.

Finally, differences in experimental design, sample size, duration of measurement, statistical models, and adjustment variables may contribute to inconsistent findings [20]. Feed efficiency and methane phenotyping are both subject to substantial individual and temporal variation, and studies with limited numbers of animals may have insufficient statistical power to detect modest biological associations. Furthermore, adjusting methane emissions for feed intake, body weight, production, or other correlated traits can substantially alter the biological interpretation of the resulting phenotype [20]. Greater harmonization of definitions, measurement protocols, reporting standards, and analytical approaches would therefore improve comparability among studies and facilitate the integration of datasets across research groups.

Taken together, these sources of heterogeneity indicate that the relationship between feed efficiency and enteric methane emissions should not be interpreted as a single universal association [20]. Rather, it emerges from interactions among the animal, diet, rumen microbiome, production environment, phenotype definition, and measurement methodology. Future studies should consequently report multiple complementary methane and efficiency phenotypes, provide detailed descriptions of diets and measurement protocols, and evaluate whether observed relationships persist across populations and production environments. Such standardization will be essential for distinguishing context-specific responses from robust biological associations and for translating research findings into effective breeding, nutritional, and management strategies.

### 4.6. Knowledge Gaps and Future Research Directions

Despite the substantial growth of research on feed efficiency and enteric methane emissions over the last decade, important knowledge gaps remain before these traits can be fully integrated into practical strategies for sustainable ruminant production. The thematic evolution and burst analyses identified in the present review indicate a clear transition toward methane mitigation, nutrient utilization, genomic approaches, rumen microbiology, and increasingly integrated assessments of animal efficiency. However, the heterogeneity of the available evidence indicates that further progress will depend not only on generating additional data but also on improving the comparability, biological interpretation, and applicability of the phenotypes being measured.

One major priority is the standardization and simultaneous evaluation of feed-efficiency and methane phenotypes. The use of different efficiency indicators and alternative expressions of methane emissions makes comparisons among studies difficult and may lead to apparently conflicting conclusions. Future studies should therefore consider reporting complementary measures of feed intake, productive performance, feed efficiency, methane production, methane yield, methane intensity, and, when appropriate, residual methane. Simultaneous characterization of these traits would provide a more comprehensive assessment of whether reductions in methane are attributable primarily to lower feed intake, greater productive efficiency, or intrinsic differences in methane production. Harmonized definitions and measurement protocols would also facilitate meta-analyses and the combination of datasets across institutions and countries [20].

Expanding the scale and diversity of phenotyping represents a second important challenge. Measurements of individual feed intake and enteric methane remain costly and technically demanding, which restricts the size of experimental populations and limits the representation of diverse production environments [14,31]. The development and validation of high-throughput and lower-cost phenotyping approaches are therefore essential for generating the large datasets required for genomic evaluation and robust biological inference. Particular attention should be given to measurements under commercial and pasture-based conditions, where animals are exposed to greater variability in diet quality, climate, grazing behavior, activity, and management than under controlled experimental conditions. Establishing whether associations identified in research facilities remain consistent under practical production conditions will be critical for their application by the livestock sector [15,17,31].

The taxonomic and production-system coverage of future research also warrants attention. The thematic patterns identified in this review indicate a strong emphasis on cattle, while the evidence available for other economically important ruminants remains comparatively less prominent. Greater representation of sheep, goats, buffaloes, and locally adapted breeds would broaden understanding of whether the biological relationships between feed efficiency and methane production are consistent across ruminant species and genetic backgrounds. This is particularly relevant because production systems, diets, climatic conditions, and selection objectives differ substantially across regions, and mitigation strategies developed for intensive cattle systems may not be directly transferable to extensive or small-ruminant production systems [4,7,9].

Another major research frontier is the integration of host genetics with rumen microbial information. The increasing prominence of metagenomics and microbial ecology in the thematic analyses suggests growing recognition that variation in feed efficiency and methane production results from interactions among the animal genome, rumen microbial community, diet, and environment [6,11,18,19,21]. Future studies should move beyond simple associations between individual microbial taxa and phenotypes toward functional characterization of microbial pathways involved in hydrogen production and utilization, fermentation, and methanogenesis [6,7,11]. Combining genomic, metagenomic, transcriptomic, metabolomic, and detailed nutritional information may help identify biological mechanisms and biomarkers capable of predicting animals that simultaneously exhibit favorable feed efficiency and methane phenotypes.

Precision livestock technologies may further accelerate this transition by enabling repeated and potentially less invasive measurements of individual animals. Automated feed-intake systems, methane-monitoring technologies, sensors, behavioral monitoring, and predictive models can generate longitudinal phenotypes that better represent temporal variation than isolated measurements [31]. When integrated with genomic and microbial information, these data may support the development of prediction tools for animals that are difficult or expensive to phenotype directly. Nevertheless, such proxy phenotypes and predictive approaches must be validated across independent populations, diets, environments, and production systems before widespread implementation.

From a genetic-improvement perspective, an important objective will be to determine how methane-related traits can be incorporated into breeding programs without compromising productivity, animal health, fertility, or other economically relevant characteristics [18,19,21]. Selection solely for reduced absolute methane production could favor animals with lower feed intake or production if the biological relationships among traits are not appropriately considered. Multi-trait selection approaches that simultaneously account for feed efficiency, methane emissions, productive performance, and other functional traits may therefore provide a more balanced strategy [18,19,21]. The development of economically and environmentally meaningful selection indexes will require robust estimates of genetic correlations and an explicit definition of whether the breeding objective is to reduce total methane production, methane per unit of feed, methane per unit of animal product, or methane production independent of intake and performance.

Finally, future research should increasingly evaluate feed efficiency and methane mitigation at the production system level. Improvements observed at the individual-animal level do not automatically translate into equivalent reductions in total environmental impact. Changes in productivity, stocking rate, replacement rate, longevity, feed production, and total output can influence the net effect of genetic or nutritional interventions. Integrating animal-level phenotypes with life-cycle and whole-system assessments would therefore provide a more complete understanding of the environmental consequences of selecting or managing animals for improved efficiency and reduced methane emissions.

Overall, the next phase of research in this field is likely to be characterized by a transition from identifying associations toward predicting and manipulating integrated efficiency–methane phenotypes. Progress will depend on standardized and large-scale phenotyping, greater representation of diverse ruminant species and production environments, and the integration of nutritional, genomic, microbial, and precision livestock information. Rather than pursuing feed efficiency and methane mitigation as independent objectives, future research should prioritize animals and production systems that optimize nutrient use and productive performance while achieving measurable and persistent reductions in environmental impact.

### 4.7. Study Limitations

Because this study was primarily designed as a bibliometric review complemented by a narrative qualitative synthesis, no formal assessment of study quality or risk of bias was performed. Therefore, the biological associations discussed should be interpreted as a narrative synthesis of the patterns reported in the retrieved literature rather than as a quantitative assessment of the strength or certainty of evidence.

In addition, the external validity of the biological patterns identified in this review is constrained by the characteristics of the available literature. The evidence base is predominantly focused on cattle, whereas sheep, goats, buffaloes, and other ruminant species remain comparatively underrepresented. Moreover, a substantial proportion of detailed feed-efficiency and methane studies rely on controlled experimental conditions, which may not fully represent commercial, extensive, or pasture-based production systems. Consequently, the associations discussed in this review should not be generalized across ruminant species, diets, genetic backgrounds, or production environments without appropriate validation under the specific conditions of interest.

## 5. Conclusions

Research addressing feed efficiency and enteric methane emissions in ruminants has expanded substantially over the last decade, reflecting the increasing convergence between animal productivity and environmental sustainability. The bibliometric evidence revealed a rapidly growing and highly collaborative research field, characterized by strong international networks and a progressive thematic transition from the characterization of feed-efficiency and methane traits toward integrated approaches involving genetics, rumen microbiology, nutrient utilization, precision phenotyping, and methane mitigation.

The available evidence indicates that improved feed efficiency can contribute to lower enteric methane emissions, particularly because more efficient animals may require less feed to achieve a given level of production. However, feed efficiency cannot be considered a universal proxy for reduced methane emissions. Associations depend on the efficiency indicator and methane phenotype evaluated, as well as on diet, animal characteristics, production system, and measurement methodology. In particular, lower absolute methane production in efficient animals does not necessarily imply lower methane production per unit of feed consumed or an intrinsically lower methanogenic potential. Therefore, future strategies should consider feed efficiency and methane emissions as complementary rather than interchangeable traits. Integrating standardized phenotyping with nutritional, genomic, microbial, and precision livestock approaches will be essential for identifying animals and production systems capable of combining efficient resource use, high productivity, and persistent reductions in enteric methane emissions.

## Figures and Tables

**Table 1:** Search strategy used for retrieving publications on feed efficiency and enteric methane emissions in ruminants.

Database	Search Strategy
Scopus	TITLE-ABS-KEY((“feed efficiency” OR “feed conversion ratio” OR “feed conversion efficiency” OR “residual feed intake” OR “residual gain” OR “residual intake and gain” OR “feed saved”) AND (methane OR “methane emission*” OR “enteric methane” OR methanogenesis OR methanogen) AND (ruminant* OR cattle OR bovine OR beef OR dairy OR sheep OR ovine OR lamb OR goat OR caprine OR buffalo*))
Web of Science	TS = ((“feed efficiency” OR “feed conversion ratio” OR “feed conversion efficiency” OR “residual feed intake” OR “residual gain” OR “residual intake and gain” OR “feed saved”) AND (methane OR “methane emission” OR “enteric methane” OR methanogenesis OR methanogen) AND (ruminant OR cattle OR bovine OR beef OR dairy OR sheep OR ovine OR lamb* OR goat* OR caprine OR buffalo))

**Table 2:** General characteristics of the studies included in the bibliometric and thematic review.

Description	Results
Main information about data	
Timespan	2016:2025
Sources (Journals, Books, etc.)	158
Documents	659
Annual Growth Rate %	13.97
Document Average Age	4.58
Average citations per doc	26.24
Document contents	
Keywords Plus (ID)	1419
Author’s Keywords	1406
Authors	
Authors of single-authored docs	11
Authors collaboration	
Single-authored docs	11
Co-Authors per Doc	6.67
International co-authorships %	40.36
Document types	
Article	536
Article; book chapter	3
Article; early access	1
Article; proceedings paper	11
Correction	1
Editorial material	2
Meeting abstract	10
Proceedings paper	6
Review	89

**Table 3:** Ten most productive authors in research on feed efficiency and enteric methane emissions in ruminants.

Author	Articles	Articles Fractionalized
Guan L.	21	5.30
Richardson C.	11	2.60
Huhtanen P.	10	2.57
Lovendahl P.	12	2.25
Lassen J.	12	2.23
Wang Y.	10	2.09
Waters S.	13	2.07
Wanapat M.	6	2
Brade W.	3	2
Amer P.	10	1.90
Zhang X.	12	1.82
Kenny D.	11	1.79
Benchaar C.	4	1.71
Miglior F.	15	1.70
Bayat A.	8	1.70
Yan T.	9	1.68
Roehe R.	12	1.66
Pryce J.	14	1.63
Lee S.	8	1.57
Beauchemin K.	9	1.56

**Table 4:** Author keywords with the strongest occurrence bursts in research on feed efficiency and enteric methane emissions in ruminants from 2016 to 2025.

Keyword	Year	Strength	Begin	End	2016–2025									
					16	17	18	19	20	21	22	23	24	25
Methane	2016	4.60	2016	2017										
Methane emission	2016	3.12	2024	2024										
Methane mitigation	2016	2.47	2024	2025										
Average daily gain	2016	2.37	2021	2022										
Residual feed intake	2016	2.14	2018	2018										
Nutrient utilization	2016	1.96	2023	2023										
Metagenomics	2016	1.85	2017	2019										
Beef cattle	2016	1.82	2024	2024										
Efficiency	2016	1.78	2021	2023										
Residual methane	2016	1.77	2021	2022										

Note: Yellow bars indicate the duration of the strongest citation burst for each keyword, while the dark background indicates periods in which no citation burst was detected.

**Table 5:** Co-cited authors with the strongest citation bursts in research on feed efficiency and enteric methane emissions in ruminants from 2016 to 2025.

Keyword	Year	Strength	Begin	End	2016–2025									
					16	17	18	19	20	21	22	23	24	25
PINARES-PATIÑO C.S.	2016	9.42	2016	2018										
HERD R.M.	2016	8.03	2016	2018										
HEGARTY R.S.	2016	7.59	2016	2018										
NKRUMAH J.D.	2016	7.45	2016	2018										
FLAY H.E.	2019	6.51	2020	2022										
ROBINSON D.L.	2016	6.28	2016	2017										
KELLY A.K.	2016	5.92	2018	2021										
KHOLIF A.E.	2018	5.91	2024	2025										
JAMI E.	2016	5.59	2017	2019										
WANG J.	2020	5.53	2025	2025										
WAGHORN G.C.	2016	5.42	2016	2018										
GOOPY J.P.	2016	4.90	2016	2017										
BELL M.J.	2016	4.78	2016	2020										
MCDONNELL R.P.	2017	4.70	2020	2022										
KNAPP J.R.	2016	4.58	2020	2022										

Note: Yellow bars indicate the duration of the strongest citation burst for each keyword, while the dark background indicates periods in which no citation burst was detected.

## Data Availability

The data supporting the findings of this study, including the bibliographic dataset used for the bibliometric and thematic analyses, are publicly available at https://drive.google.com/uc?export=download&id=1TmbtDy0QyrqXTS-OPivD5zVwhOrvM1Ok (accessed on 20 July 2026).
